# Trace fossils on dinosaur bones reveal ecosystem dynamics along the coast of eastern North America during the latest Cretaceous

**DOI:** 10.7717/peerj.4973

**Published:** 2018-06-11

**Authors:** Chase D. Brownstein

**Affiliations:** Research Associate, Stamford Museum and Nature Center, Stamford, CT, United States of America

**Keywords:** Taphonomy, Cretaceous, Dinosaurs, Appalachia, Theropods, Crocodyliforms

## Abstract

Direct evidence of paleoecological processes is often rare when the fossil record is poor, as in the case of the Cretaceous of eastern North America. Here, I describe a femur and partial tibia shaft assignable to theropods from two Late Cretaceous sites in New Jersey. The former, identifiable as the femur of a large ornithomimosaur, bears several scores interpreted as shark feeding traces. The tibia shaft has punctures and flaked bone from the bites of mid-sized crocodyliforms, the first documented occurrence of crocodyliform traces on dinosaur bone from the Maastrichtian of the Atlantic Coastal Plain. The surface of the partial tibia is also littered with indentations interpreted as the traces of invertebrates, revealing a microcosm of biological interaction on the coastal seafloor of the Cretaceous Atlantic Ocean. Massive crocodyliforms, such as *Deinosuchus rugosus* and the slightly smaller *Deltasuchus motherali*, maintained the role of terrestrial vertebrate taphonomic process drivers in eastern North America during the Cretaceous. The report of crocodyliform bite marks on the ornithomimosaur tibia shaft in this manuscript reinforces the importance of the role of crocodyliforms in the modification of terrestrial vertebrate remains during the Cretaceous in North America. The preserved invertebrate traces add to the sparse record of the presence of barnacles and other marine invertebrates on dinosaur bone, and the evidence of shark feeding on the ornithomimosaur femur support the “bloat-and-float” model of terrestrial vertebrate fossil deposition in marine deposits from the Cretaceous of eastern North America.

## Introduction

Crocodyliform bite marks on vertebrate remains are well-represented throughout the Mesozoic and Cenozoic and have been extensively described (e.g., Carpenter & Lindsey, 1980; Binford, 1981; Erickson, 1984; Davidson & Soloman, 1990; Schwimmer, 2002; Forrest, 2003; Fuentes, 2003; Cisneros, 2005; Milukas et al., 2006; Njau & Blumenschine, 2006; Schwimmer, 2010; Noto, Main & Drumheller, 2012; Boyd, Drumheller & Gates, 2013; Martin, 2013; Drumheller & Brochu, 2014; Drumheller & Brochu, 2016; Njau & Gilbert, 2016). Despite a poor fossil record, one pattern that has emerged in the study of the paleoecology of eastern North America during the Cretaceous is the frequency of vertebrate remains—especially those of turtles and dinosaurs—that show evidence of feeding by crocodyliforms. Such marks have been documented in fossils from the Cenomanian Woodbine Formation of Texas and attributed to the bites of individuals of the taxon *Deltasuchus motherali* (Noto, Main & Drumheller, 2012; Adams, Noto & Drumheller, 2017). In the Campanian, evidence of crocodyliform feeding on dinosaurs and turtles from multiple geological units in the southeastern United States and from the Marshalltown Formation of New Jersey have been attributed to the massive crocodylian *Deinosuchus rugosus*, a species populous along the eastern North American coastline during that time (e.g., Schwimmer et al., 1993; Gallagher, 1995; Schwimmer, 1997; Schwimmer, 2002; Schwimmer, 2010; Schwimmer et al., 2015). However, no record of large crocodyliform bite marks on dinosaur material has been reported from the Maastrichtian of eastern North America, when *Deinosuchus* disappears from the fossil record (Schwimmer, 2002). In the Maastrichtian of the Atlantic Coastal Plain, crocodyliforms are represented by various forms smaller than *D. rugosus*, including *Borealosuchus threeensis*, *Thoracosaurus neocesariensis*, *Hyposaurus rogersii*, and *Elosuchus minor* (De Kay, 1842; Carpenter, 1983; Parris, 1986; Gallagher, 1993; Brochu, 2006; Brochu et al., 2012). Among these, *Borealosuchus threeensis* and *Thoracosaurus neocesariensis* seem to have grown the largest; individuals of the both taxa achieved sizes of 5 or more meters, and one *Thoracosaurus* specimen may have reached 7–8 m in length (e.g., Schwimmer, 2002; Brochu et al., 2012).

Like crocodyliform feeding traces, shark feeding traces are also extensively documented in the literature (Everhart, Everhart & Shimada, 1995; Schwimmer, 1997; Everhart, 1999; Shimada & Everhart, 2004; Shimada & Hooks, 2004; Everhart & Ewell, 2006; Boessenecker & Perry, 2011; Schein & Poole, 2014; Hill et al., 2015). Traces on dinosaur bones from sharks are predictably common the Campanian and Maastrichtian of eastern North America, owing to the preservation of non-avian dinosaurs from eastern North America in marine strata. Such finds include the heavily shark-bitten partial femur of a diminutive adult hadrosaurid from the Hornerstown Formation (Schein & Poole, 2014) and other remains of hadrosaurids, nodosaurids, and tyrannosauroids with characteristic scores (e.g., Carpenter, Dilkes & Weishampel, 1995; Schwimmer, 1997; Schwimmer, Stewart & Williams, 1997; Everhart & Ewell, 2006; Brownstein, 2017). Some of these occurrences of shark feeding traces on dinosaur bones may have been caused by individuals of the medium-sized species *Cretolamna appendiculata*, and there is direct evidence (embedded teeth) to show that *Squalicorax kaupi* occasionally scavenged dinosaur bone (e.g., Schwimmer, 1997; Schwimmer, Stewart & Williams, 1997; Schein & Poole, 2014). These shark feeding traces on dinosaur bones have been noted in the study of eastern North American dinosaur taphonomy to support the prevalence of the “bloat and float” hypothesis in eastern North American dinosaur preservation (Langston, 1960; Bryan et al., 1991; Schwimmer, 1997; Schwimmer, 2002), whereby dinosaur carcasses washed out to sea, remained buoyant in the water due to an internal buildup of gas, and slowly lost body parts that would become fossilized on the sea floor.

Invertebrate traces on dinosaur bones are somewhat uncommon, though insect traces on bones deposited in inland settings have been extensively described in the literature (e.g., Rogers, 1992; Hasiotis, Fiorillo & Hanna, 1999; Paik, 2000; West & Martin, 2002; Hasiotis, 2004; Kirkland & Bader, 2010; Roberts, Rogers & Foreman, 2007; West & Hasiotis, 2007; Bader, Hasiotis & Martin, 2009; Xing et al., 2016). These vary in morphology (e.g., Bader, Hasiotis & Martin, 2009; Xing et al., 2016) and have been shown as important indicators of the taphonomy of the bones on which they lie (e.g., Martin & West, 1995; Hasiotis, Fiorillo & Hanna, 1999; West & Hasiotis, 2007; Bader, Hasiotis & Martin, 2009).

A variety of Mesozoic-age invertebrate traces on terrestrial and marine vertebrates, including those of the polychaete *Osedax* and associations of pelycopods, molluscs, have also been described (e.g., Martill, 1987; Grange & Benton, 1996; Maier, 2003; Kaim et al., 2008; Buckeridge, 2011; Danise & Higgs, 2015). Additional occurrences of invertebrate traces on marine vertebrates include records on mammal and penguin bones (e.g., Frenguelli, 1928; Deméré & Cerutti, 1982; Kues, 1983; Donovan, 1988; Cigala-Fulgosi, 1990; Emslie et al., 1996; Hulbert et al., 1998; Hoyle et al., 2004; Cione et al., 2010; Kiel et al., 2010; Boessenecker, 2013; Boessenecker & Fordyce, 2015). In the Maastrichtian of the Atlantic Coastal Plain, invertebrate borings are common on the fossil shells of the bivalves *Exogyra costata* and *Pycnodonte mutabilis* and are attributed to the sponge *Cliona cretacica* (e.g., Fenton & Fenton, 1932). Marine invertebrate traces on vertebrate bones have allowed for the reconstruction of poorly-known Mesozoic benthic ecosystems and the origin of modern oceanic deadfall flora and fauna (e.g., Danise & Higgs, 2015).

Here, I describe two theropod dinosaur bones from the Maastrichtian of New Jersey. One is a partial tibia shaft, the other the distal end of a femur. The partial femur is assignable to an as-yet-unrecognized large ornithomimosaur of similar size to the Asian taxon *Gallimimus* and an unnamed animal from the Campanian Dinosaur Park Formation of Alberta (Longrich, 2008). Both New Jersey specimens show marks attributable to feeding, the tibia shaft bearing ones from crocodyliforms and the femur from sharks, allowing for insight into the faunal composition and paleoecology of Maastrichtian communities along the coast of the Cretaceous Atlantic Ocean (Fiorillo, 1991; Gallagher, 1995; Schwimmer, 1997; Chure, Fiorillo & Jacobsen, 1998; Schwimmer, 2002; Rogers, Krause & Rogers, 2003; Jennings & Hasiotis, 2006; Reisz & Tsuji, 2006; Schwimmer, 2010; Noto, Main & Drumheller, 2012; Main, Noto & Weishampel, 2014; Adams, Noto & Drumheller, 2017). At least two morphotypes of invertebrate traces are also present on the dinosaur tibia, including those tentatively identified as barnacle scars that represent, to the author’s knowledge, only the second occurrence of these encrusting organisms on non-avian dinosaur bone (Maier, 2003; Boessenecker, 2013).

## Materials & Methods

### Permits

No permits were needed for this study, and access to the collections of the Peabody Museum of Natural History was provided by Daniel Brinkman.

### Geological Setting

In the 1970s, two partial theropod hindlimb bones were recovered from two Cretaceous-age sites in New Jersey by Gerard R. Case and Ralph O. Johnson. The tibia shaft portion YPM VPPU.021825 was collected from Maastrichtian deposits at the Big Brook site in Monmouth County, New Jersey. There has been some debate as to the exact provenance of the majority of the fossils from this locality that are eroded from sediments along the riverbanks (e.g., Lauginiger, 1986; Becker & Chamberlain, 2001; Gallagher et al., 2014), but recent studies have found that most specimens of Late Cretaceous dinosaurs from the site are from the early to mid-Maastrichtian Navesink Formation (e.g., Miller et al., 2004; Brusatte et al., 2012). The partial distal femur YMP VPPU.022361 was recovered from the Navesink Formation at Hop Brook near Holmdel, New Jersey (Baird, 1986).

The environment represented by the Navesink Formation (69–67 Ma; Miller et al., 2004) at Big Brook is marine in origin, representing a transgression of the Atlantic Ocean (e.g., Gallagher, Parris & Spamer, 1986; Lauginiger, 1986; Gallagher, 1993; Weishampel & Young, 1996; Miller et al., 2004; Parris, Grandstaff & Gallagher, 2004). The Navesink Formation at Big Brook represents the deepest, most saline environment at the locality and is highly fossiliferous at some intervals (Gallagher, Parris & Spamer, 1986). Terrestrial vertebrate fossils from the site include the worn bones of lambeosaurines and indeterminate hadrosaurids, nodosaurids, tyrannosauroids, and ornithomimosaurs (e.g., Gallagher, Parris & Spamer, 1986; Gallagher, 1993; Weishampel & Young, 1996; Brusatte et al., 2012). The marine vertebrate fauna is extensive and includes the crocodyliform *Thoracosaurus*, several different species of turtles and mosasaurs, and a menagerie of chondrichthyan and osteichthyan taxa (e.g., Gallagher, Parris & Spamer, 1986; Lauginiger, 1986; Gallagher, 1993).

### Identification and documentation of traces

The surfaces of both bones were extensively searched for fossil traces. Artifacts of preparation were carefully identified and excluded. The presence of any preparation artifacts potentially interpretable as trace fossils is unlikely, as both YPM VPPU.021825 and YPM VPPU.022361 were collected from the surface after being eroded out of Cretaceous exposures on the banks of Big Brook and Hop Brook. Probable traces were reviewed, photographed, and measured using digital calipers. The width of each trace was taken along each’s midway, with length measured along greatest axis of each bone.

The nomenclature of Binford (1981) was used for the vertebrate traces described herein. Crocodyliform traces were identified based on the criteria of Njau & Blumenschine (2006) and through comparisons with other descriptions of fossil crocodyliform feeding traces in the literature. Shark feeding traces on the described femur were identified based on their identical nature to the arced scores on vertebrate bones identified as shark traces in previous studies (e.g., Everhart, Everhart & Shimada, 1995; Schwimmer, 1997; Everhart, 1999; Shimada & Everhart, 2004; Shimada & Hooks, 2004; Everhart & Ewell, 2006; Boessenecker & Perry, 2011; Schein & Poole, 2014; Hill et al., 2015). Traces referred to invertebrates are called “small biological traces” in the descriptive section of this manuscript and referred to specific clades in the Discussion section based on comparisons with other marine invertebrate traces on vertebrate bones documented from the fossil record (e.g., Frenguelli, 1928; Deméré & Cerutti, 1982; Kues, 1983; Donovan, 1988; Cigala-Fulgosi, 1990; Emslie et al., 1996; Hulbert et al., 1998; Hoyle et al., 2004; Cione et al., 2010; Kiel et al., 2010; Boessenecker, 2013; Boessenecker & Fordyce, 2015).

## Systematic Paleontology

**Table:** 

Dinosauria Owen (1842) sensu Padian & May (1993)
Theropoda Marsh (1881) sensu Gauthier (1986)
Theropoda indet.

Material: YPM VPPU.021825, partial tibia shaft.

Referral: The tibia may be attributed to a theropod based on its hollow interior.

**Figure 1:**
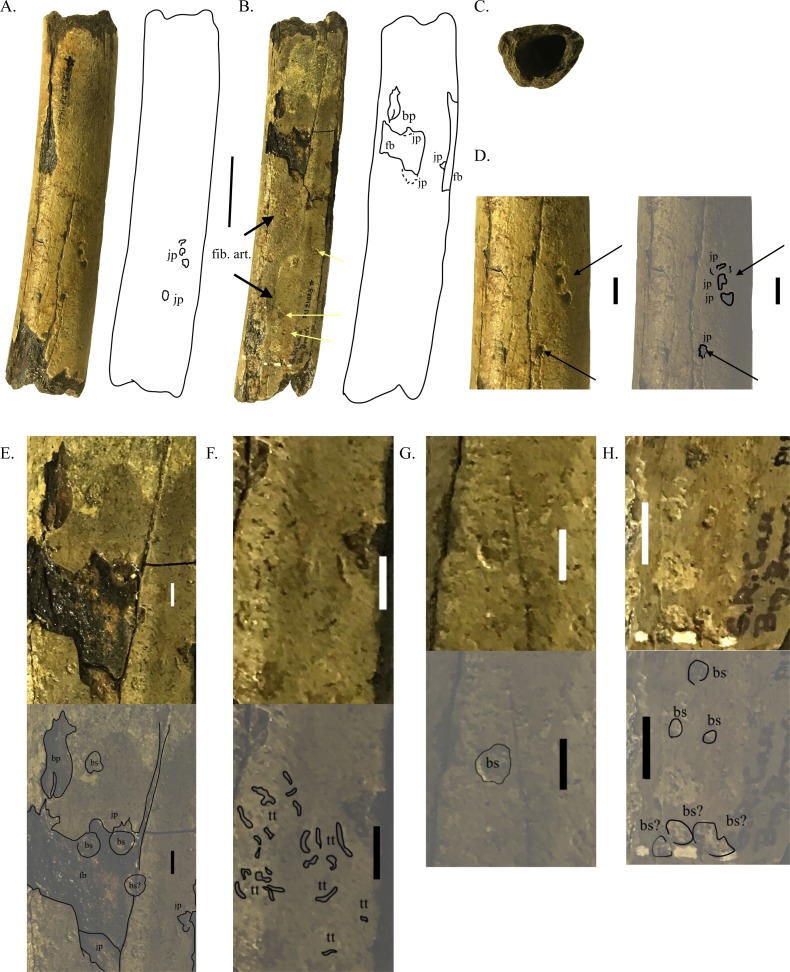
Partial tibia shaft with crocodyliform feeding marks and invertebrate traces. YPM VPPU.021825 in lateral (A), medial (B), and proximal (C) views, with closeups of crocodyliform feeding marks and possible invertebrate burrows on the lateral (D) and medial (E–I) faces of the bone. Scale bar =50 mm (A–C), 5 mm (D–I). Black arrows indicate crocodyliform feeding marks; yellow arrows indicate possible invertebrate traces. Abbreviations: bp, bisected puncture; bs, barnacle scar; fb, flaked bone; jp, jagged puncture; tt, tubular traces.

Description: YPM VPPU.021825 (Figs. 1A–1E) is the partial tibia shaft of a large theropod dinosaur (e.g., Baird, 1986; Gallagher, 1993). The bone still preserves a poorly developed articular surface for the fibula on its medial surface that is bordered by two slight, proximodistally-running ridges. The bone is straightened and, in cross-sectional view, has a greater dorsoventral than mediolateral width. In size, the tibia shaft compares most favorably with ornithomimosaur and tyrannosaur specimens collected from the Atlantic Coastal Plain, and thus it is likely the bone came from one of these two groups of theropod dinosaur. Measurements of this specimen may be found in Table 1.

**Table:** 

Dinosauria Owen (1842) sensu Padian & May (1993)
Theropoda Marsh (1881) sensu Gauthier (1986)
Coelurosauria Von Huene (1914) sensu Sereno, McAllister & Brusatte (2005)
Ornithomimosauria (Barsbold, 1976) sensu Choiniere, Forster & De Klerk (2012)
Ornithomimosauria indet.

Material: YPM VPPU.022361, partial distal left femur.

Referral: The femur may be tentatively assigned to Ornithomimosauria based on a combination of morphological features, as it was too incomplete to be included in a phylogenetic analysis. YPM VPPU.022361 is assigned to Ornithomimosauria based sharing with femora from taxa of this clade its (1) elongate nature, which was originally used by Baird (1986) for this assignment, (2) the presence of a thin crest extending proximally from the distal medial condyle, and (3) heavily separated distal condyles (e.g., Makovicky, Kobayashi & Currie, 2004). Besides ornithomimosaurs, only dromaeosaurids and tyrannosauroids are known from the Campanian-Maastrichtian of Appalachia (e.g., Baird & Horner, 1979; Gallagher, 1993; Weishampel & Young, 1996; Kiernan & Schwimmer, 2004; Carr, Williamson & Schwimmer, 2005; Brusatte, Benson & Norell, 2011; Brusatte et al., 2012; Schwimmer et al., 2015). All described dromaeosaurids from Appalachia are smaller than the theropod to which the YPM specimen described herein belongs (Kiernan & Schwimmer, 2004; Schwimmer et al., 2015), and dromaeosaurids of similar size to the dinosaur that the Big Brook femur represents have more robustly built femora with only slightly separated distal condyles and without a distal medial ridge (e.g., Norell & Makovicky, 2004). The femur is also differentiated from tyrannosauroids like *Dryptosaurus* and *Appalachiosaurus* based on the features noted above (fig. 16A–D in Carr, Williamson & Schwimmer (2005); fig. 15 in Brusatte, Benson & Norell, (2011)).

**Figure 2:**
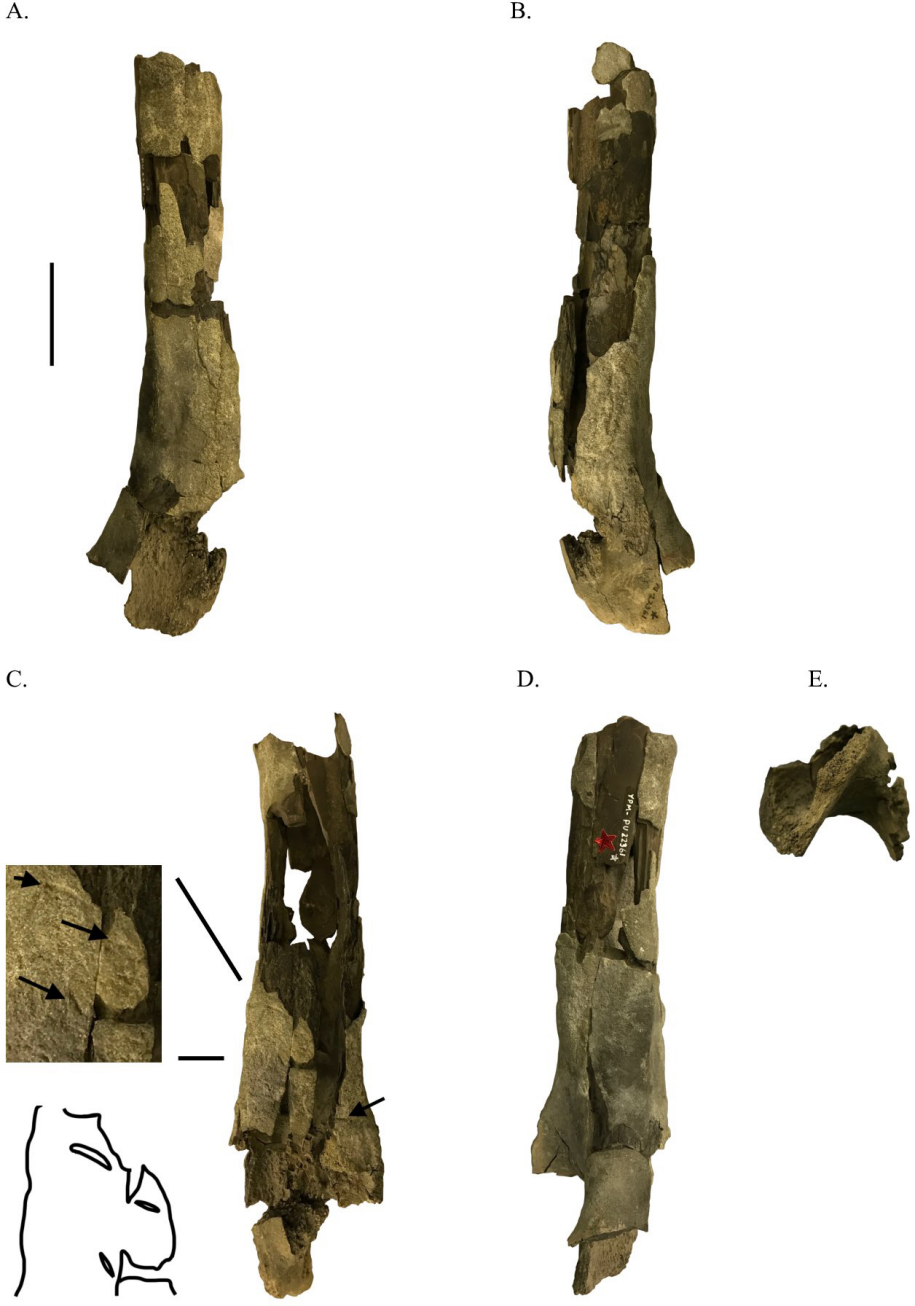
Distal ornithomimosaur femur with shark feeding scores. YPM VPPU.022361 in lateral (A), medial (B), dorsal (C) ventral (D), and distal (E) views. Scale bar =5 mm. Black arrows indicate shark feeding scores.

Description: YPM VPPU.022361 (Figs. 2A–2F) is the distal femur of a large ornithomimosaur. The specimen is of comparable size to the femora of *Gallimimus* (Table 1; Osmólska, Roniewicz & Barsbold, 1972; Baird, 1986) and is hollow. Portions of both the lateral and medial distal femoral condyles are preserved and are separated by a prominent intercondylar groove. On the medial surface of the medial distal condyle, a ridge originates that extends proximodorsally. The femur is slightly divergent dorsally towards its distal end in medial and lateral views. Muscle attachment scars are present on the preserved portion of the lateral surface of the bone.

**Table 1:** Measurements of theropod bones.

Specimen	Proximodistal length	Distal mediolateral width	Distal dorsoventral width	Circumference at midshaft	Reference
YPM VPPU.021825	222 mm	n/a	n/a	152 mm	this paper
YPM VPPU.022361	290 mm	75 mm	59 mm	149 mm	Baird (1986), this paper

### Remarks on the traces present on the theropod limb bones

YPM VPPU.021825: The lateral surface of the bone preserves four jagged punctures, including three that are adjacent to each other and may represent a single biting event based on their similar size, closely adjacent nature, and placement matching the curved mesial end of the tooth row of a squarish skull (Fig. 1D). Of these serial bite marks, the distal is the largest and most rounded, with the jagged punctures proximal to the largest one curving towards the dorsal surface. This indicates that the serial bite marks were left by mesial dentition corresponding to the curvature of the anterior end of a squarish vertebrate jaw. Distal to all these punctures on the bone, a single jagged pit is also present. These punctures penetrate appreciably into the bone surface, and their measurements are catalogued in Table 2.

On the medial surface of YPM VPPU.021825, four punctures are preserved (Figs. 2B, 2E). One of these is a jagged puncture, proximally adjacent to the edge of a major area of flaked bone and laterally and dorsally to major breaks in the bone surface (Figs. 1B, 2E). The largest, an elongate, deepened bisected puncture, sits within the distal portion of smaller area of spalled bone (Figs. 1B, 2E). Two other jagged punctures appear along the borders of major areas of bone flaking on the medial and ventral ends of the tibia shaft (Fig. 1E). Because these areas of bone flaking are adjacent to these punctures, they likely were created by the same biting events (e.g., Njau & Blumenschine, 2006; Njau & Blumenschine, 2012; Drumheller & Brochu, 2014; Njau & Gilbert, 2016; Drumheller & Brochu, 2016). Along with those from the punctures on the lateral surface, measurements of those present on the medial surface are catalogued in Table 2.

**Table 2:** Measurements of crocodyliform and shark feeding traces.

Bite mark	Length	Width
Proximalmost lateral (YPM VPPU.021825)	3 mm	1 mm
Second proximalmost lateral (YPM VPPU.021825)	4 mm	3 mm
Second distalmost lateral (YPM VPPU.021825)	5 mm	4 mm
Distalmost lateral (YPM VPPU.021825)	4 mm	3 mm
Bisected medial (YPM VPPU.021825)	10 mm	6.5 mm
Larger jagged medial (YPM VPPU.021825)	6 mm	5 mm
Smaller jagged medial (YPM VPPU.021825)	?	6 mm
Jagged ventral (YPM VPPU.021825)	?	5 mm
Proximalmost (YPM VPPU.022361)	9 mm	1 mm
Second proximalmost (YPM VPPU.022361)	6 mm	1 mm
Middle (YPM VPPU.022361)	5 mm	0.5 mm
Distalmost (YPM VPPU.022361)	4 mm	1 mm

The medial surface of the partial tibia shaft YPM VPPU.021825 is littered with two morphotypes of rounded, nearly radially and bilaterally symmetrically outlined shapes interpreted as biological traces. These shapes are unlike those expected from artifacts of erosion, which would be comparatively jagged and appear as heavy abrasions, rather than small, detailed stains and indentations, on the bones. The majority of these inferred biological traces on the surface of this bone appear as tubular indentations that are oriented sub-parallel to the longitudinal axis of the bone and do not extend past the cortical bone layer.

One morphotype of small biological trace (Fig. 2E) appears as elongate, ovoid to circular traces that are heavily clustered (*n* > 40) between to the large bone flake on the medial surface of the tibia and the bone flake on the ventral surface of the bone. These are very shallow traces that penetrate slightly more into the bone than the larger ovoid small biological traces described below. The longest of these traces is approximately 2 mm long and 0.20 mm wide, whereas the smallest is less than 0.1 mm long.

Another morphotype of trace appears as circular shapes that forms stains or extremely shallow depressions present across the medial surface of the tibia shaft (Figs. 1E–1G). These stains are considerably darker than the outside bone surface and are identified as biological traces based on their highly circular shape. Several of these traces are present on the medial surface of the bone, three of which are present as stains sitting within the flaked bone associated with the punctures described above. A number of these circular traces are also present as shallow depressions on the distal end of the bone, whereas only one is located near the diaphysis of the partial tibia shaft (Figs. 1F–1G). The majority of these traces are approximately 4 mm in diameter.

YPM VPPU.023361: At least four gently arched scores are present on the distal ornithomimosaur femur YPM VPPU.023361 (Fig. 2C). None of these scores are paired and each clearly came from only one tooth cusp, none bear serration striations, and all are deepened, varying slightly in width at their midpoints. The scores are concentrated on the ventral surface of the distal femur YPM VPPU.023361, although several scrapes on the lateral and dorsal surfaces may also be feeding traces. Measurements of these marks may be found in Table 2.

## Discussion

### Assignment of traces on the described bones to specific clades

The possibilities that the large, deepened punctures and major patches of bone spalling on the theropod tibia YPM VPPU.021825 are those of theropod dinosaurs, mammals, plesiosaurs, or mosasaurs, which are all represented in the Navesink (e.g., Gallagher, 1993), are rejected based on several lines of evidence. Firstly, these bite marks were by an organism that may have possessed unserrated teeth due to the lack of corresponding striations near or within any of the punctures. Secondly, the deepened, extensive nature of the punctures and spalled bone are consistent with the trace maker possessing a powerful bite and possessing incrassate, rather than ziphodont, dentition. This first observation suggests against theropod dinosaurs being the inflictors of the marks, whose serrated teeth often leave striations on bone (e.g., Fiorillo, 1991; Horner & Lessem, 1993; Erickson & Olson, 1996; Carpenter, 1998; Chure, Fiorillo & Jacobsen, 1998; Jacobsen, 2001; Hyslop & Boyd, 2004; Fowler & Sullivan, 2006; Longrich & Ryan, 2010; Noto, Main & Drumheller, 2012; De Valais, Apesteguía & Garrido, 2012; Xing et al., 2012; Boyd, Drumheller & Gates, 2013; Hone & Tanke, 2015), and the second eliminates the Maastrichtian theropod dinosaurs present in the Atlantic Coastal Plain that were large enough to produce the marks on YPM VPPU.021825, tyrannosauroids, as Appalachian tyrannosauroids had heavily ziphodont, serrated teeth and relatively lightly built skulls (e.g., Brusatte, Benson & Norell, 2011). These teeth, even if contact with the bone surface was achieved along their apical end, would not produce the large, deep, rounded punctures present on the partial theropod limb shaft.

Mammals are also eliminated as agents of the punctures and flaking on YPM VPPU.021825. Firstly, the size of the animal that YPM VPPU.021825 represents is clearly much larger than non-avian dinosaurs that preserve evidence of mammalian feeding on their bones (e.g., Hu et al., 2005). Secondly, the bisected pit on the ventral surface of the tibia shaft is inconsistent with a mammalian feeding trace (e.g., Njau & Blumenschine, 2006; Boyd, Drumheller & Gates, 2013; Njau & Gilbert, 2016).

These marks are also incongruent with the traces of mosasaur bites, which are deepened and often linear in shape (e.g., Bell & Martin, 1995; Lingham-Soliar, 1998; Lingham-Soliar, 2004; Everhart, 2008; Einarsson et al., 2010). The serial bite marks present on the lateral surface of the bone suggest a squarish built for the mesial end of the jaw of the trace maker, which conflicts with the triangular morphology of the mesial end of the skull of mosasaurs (Everhart, 2008). The presence of the bisected pit and associated extensive bone flaking on the lateral surface of YPM VPPU.021825 are unlike the condition seen in mosasaur bites, where localized, heavily deepened gouges and circular punctures indicate the sharpened, rather than blunt, nature of the apical end of the teeth (e.g., Schwimmer, 2010). Only globidensine mosasaurs are known to have possessed apically blunt teeth (Schwimmer, 2010). However, these mosasaurs had weaker bite forces than eusuchian crocodyliforms and other taxa that could inflict the extensive bone damage seen on the tibia shaft, which precludes the identification of these large marine squamates as the trace makers (Schwimmer, 2002; Schwimmer, 2010). Plesiosauroid feeding traces are shallow, linear scrapes that lend to the comparatively weak bite forces of these marine reptiles (e.g., Martin, Rothschild & Burnham, 2016). The slender teeth of plesiosaurs also do not match with the large, rounded marks seen on the tibia and could not have inflicted them, let alone the extensive bone flaking on the tibia that likely occurred under high stress. Although pliosauroid plesiosaurs are known to have inflicted catastrophic bone damage to prey (e.g., Thulborn & Turner, 1993), this group is only known to have survived into the Turonian in North America (Schumacher, Carpenter & Everhart, 2013). The bisected pit on the tibia, which is diagnostic of crocodyliform bites (Njau & Blumenschine, 2006), also precludes referral of the punctures and flaking to bites from these marine reptile groups.

The punctures and areas of flaked bone on YPM VPPU.021825 satisfy three of the five criteria of Njau & Blumenschine (2006) for the identification of crocodyliform bite marks: the rarity of crocodyliform bite marks in the assemblage (only the metatarsal described herein has been noted as possessing such punctures out of the dozens of dinosaur specimens collected from Big Brook; Gallagher, 1993; C Brownstein, pers. obs., 2017), the presence of bisected punctures, and the lack of evidence for gnawing on the bone. Additionally, the marks may satisfy the criterion of Njau & Blumenschine (2006) for crocodyliform bite mark identification that the marks are populous on bones useful for leverage, though not enough are present on the preserved portion of the tibia to definitively state so. The fragmentary nature of YPM VPPU.021825 is interpreted as a taphonomic relic from erosion and deposition at sea rather than an indication of the type of organism that left the punctures and flaking on its surface (e.g., Njau & Blumenschine, 2006; Boyd, Drumheller & Gates, 2013), as other theropod bones from the Atlantic Coastal Plain bearing feeding traces clearly attributable to large crocodyliforms are also fragmentary limb shafts (e.g., Schwimmer, 2002; Schwimmer, 2010).

Although the puncture marks bear resemblance to the punctures described as *Nihilichnus nihilichnus* by Milukas et al. (2006) and Late Cretaceous-age traces referred to this ichnotaxon by Jacobsen & Bromley (2009), assignment of the traces described herein to this taxon is not effected herein. Specific assignment is not made in the context of previous studies of crocodyliform traces on dinosaur bones, which have made efforts to identify the crocodyliform morphotypes or taxa that made such traces but did not assign to specific ichnotaxa the traces themselves (e.g., Schwimmer, 2002; Rivera-Sylva, Frey & Guzmán-Gutiérrez, 2009; Schwimmer, 2010; Boyd, Drumheller & Gates, 2013).

Only one crocodyliform has been reported from the Navesink Formation: *Thoracosaurus neocesariensis* (De Kay, 1842; Gallagher, 1993; Schwimmer, 2002). At least one known specimen of this taxon reached a length of ∼7–8 m (Schwimmer, 2002). However, the deepened punctures and extensive bone spalling on the metatarsal YPM VPPU.021825 are inconsistent with the morphology of the conical, elongate, slightly hooked teeth of *Thoracosaurus* (e.g., Brochu, 2004). As the Navesink Formation represents the deepest marine environment out of the units present at Big Brook, the possibility that a previously undetected taxon of crocodyliforms living inland or along the coast inflicted such marks is certainly possible. Whatever taxon or taxa of crocodyliform inflicted the feeding traces on YPM VPPU.021825, they possessed the ability to prey or scavenge on dinosaurs of more than 3 m in length and cause extensive damage to dinosaur bones (Fig. 1).

Regarding the scores present on the femur, similar scores on dinosaur and other vertebrate bones deposited in marine settings have been attributed to sharks (e.g., Everhart, Everhart & Shimada, 1995; Schwimmer, 1997; Everhart, 1999; Shimada & Everhart, 2004; Shimada & Hooks, 2004; Everhart & Ewell, 2006; Schein & Poole, 2014; Hill et al., 2015). Though no teeth are imbedded in YPM VPPU.022361, the morphology of the scores is highly consistent with those on dinosaur bones with embedded shark teeth (e.g., Schwimmer, 1997), warranting their identification as shark feeding traces. These marks are also not mammalian or dinosaurian in origin because they lack striation marks and are inconsistent with mammalian gnawing. Only one type of bony fish present in the Navesink Formation, *Xiphactinus* (Gallagher, 1993), is similar to the size of the trace maker for the scores present on the femur. However, the teeth of this taxon are elongate and conical and do not fit with the deepened scores on the femur, which indicate sharpened, mediolaterally compressed objects created them. Because of the near-identical morphology of the scrapes on the femur described herein and previously reported shark feeding traces on vertebrate bones (Everhart, Everhart & Shimada, 1995; Schwimmer, 1997; Everhart, 1999; Shimada & Everhart, 2004; Shimada & Hooks, 2004; Everhart & Ewell, 2006; Schein & Poole, 2014; Hill et al., 2015), the most parsimonious conclusion is that the scores were inflicted by shark teeth. Gallagher (1993) reported two genera of shark in the Navesink Formation. Individuals of *Squalicorax pristodontus* may be eliminated as candidates for the bite marks on YPM VPPU.022361, as the teeth of that taxon were serrated and would have left striations on the femur (e.g., Schein & Poole, 2014). Beyond this elimination, any confident assignment of these traces to a specific shark taxon is impossible. The protocol of previous papers on shark traces from the Cretaceous of eastern North America is followed, and thus these scores are not assigned to specific taxa (e.g., Schwimmer, 1997; Schein & Poole, 2014).

The two small biological trace morphotypes on the tibia described herein are regarded as invertebrate traces. Elongate borings on a plesiosaur bone from New Jersey may be from an invertebrate similar to *Lithophaga* (R Johnson, pers. comm., 2018), which is known to burrow into corals and stromatolites (e.g., Jones & Pemberton, 1988; Akpan, 1991). *Lithophaga ripleyana* is the species known from the Navesink Formation (e.g., Gallagher, Parris & Spamer, 1986). However, *Lithophaga* leaves larger, deeper clavate borings than the elongate traces on the tibia shaft described herein. The source of these small biological traces on the tibia is thus considered an indeterminate invertebrate and remains something of a mystery.

The shallow, highly circular stains and indentations on the tibia shaft are also interpreted as invertebrate traces, specifically barnacle marks. As noted, the biological nature of both the stains and indentations is based on their notable radial or bilateral symmetry, which would be unexpected results of the modification of the bone by debris in the water as the partial limb bone was deposited. These traces compare favorably with the circular, stained barnacle attachment scars described on other fossilized vertebrate remains (e.g., Martill, 1987; Donovan, 1988; Maier, 2003; Buckeridge, 2011; Boessenecker, 2013) and are the second reported occurrence of these invertebrate traces on dinosaur bone. Both morphotypes of traces identified as those of invertebrates are nearly absent from the lateral surface of YPM VPPU.021825, indicating the medial surface was exposed to the water column and the lateral surface was buried in the substrate. However, these traces are also not assigned to specific ichnotaxa due to (1) their eroded nature and (2) the lack of described barnacle traces from the Mesozoic. Previous work on Mesozoic-age barnacle traces have also taken this tentative position regarding specific assignment of traces (e.g., Janssen, Baal & Schulp, 2013).

### Taphonomy of the dinosaur bones

The presence of the several different traces on the dinosaur specimens described herein is important in illuminating both the taphonomy of terrestrial vertebrate remains in the Maastrichtian marine deposits of the Atlantic Coastal Plain and the paleoecology of the near-shore environments of the region. The bite marks of mid-sized crocodyliforms (in comparison to the estimated sizes of other crocodyliforms to which bite marks have been assigned; e.g., Noto, Main & Drumheller, 2012; Boyd, Drumheller & Gates, 2013) on the partial tibia shaft YPM VPPU.021825 may suggest the specimen first underwent some taphonomic event in a near-shore environment before transport onto the sea floor. In Texas, an attritional vertebrate assemblage likely created by the large crocodyliform *Deltasuchus motherali* has been documented at the Arlington Archosaur site of the Cenomanian Woodbine Formation, which preserves a near-shore environment (e.g., Noto, Main & Drumheller, 2012; Adams, Noto & Drumheller, 2017). It is certainly possible that such an event occurred in the taphonomy of YPM VPPU.021825 before it was washed into the Atlantic Ocean. Once deposited at sea, the medial surface of the fragmented tibia YPM VPPU.021825 likely faced into the water column to experience significant abrasion on account of the invertebrates. The rounded state of the edges of YPM VPPU.021825 is consistent with the bone being eroded at sea and deposited in the deep, marine environment represented by the Navesink Formation rather than being reworked from older units. Furthermore, the lack of dinosaur remains from the early-middle Campanian and latest Maastrichtian-Paleogene formations exposed at Big Brook (e.g., Gallagher, 1993) suggests YPM VPPU.021825 originated in a Campanian-Maastrichtian to Maastrichtian horizon.

The distal femur YPM VPPU.022361 seems to have undergone a longer period of erosion at sea based on its rough, exfoliated surface and the presence of shark feeding traces on the bone. Several features of YPM VPPU.022361 support the prevalence of the “bloat-and-float” model among Maastrichtian terrestrial vertebrate remains in the Atlantic Coastal Plain. These include (1) the identification of the bone as the distal portion of a limb bone, (2) the presence of shark feeding traces on the bone, and (3) the bone’s eroded, fragmentary state. These taphonomic artifacts are also consistent with the preservation of the bone in the deep marine setting of the Navesink Formation.

These bones thus support the presence of two taphonomic models among terrestrial vertebrate remains in the Navesink Formation. The first includes taphonomic events in near-shore environments, such as predation or scavenging by crocodyliforms and other carnivores, and later deposition and taphonomic processes from both biotic (possible indeterminate invertebrate traces) and abiotic (water erosion) on the seafloor. The second is the “bloat-and-float” model, whereby dinosaur skeletons are washed out to sea and bones on the fringes of the skeleton fall to the sea floor and experience significant water wear, with scavenging by marine predators occurring throughout the process.

### Implications for the Maastrichtian vertebrate fauna of the Atlantic Coastal Plain

In addition to their taphonomic significance, the dinosaur femur and crocodyliform traces on the tibia described herein also add to the current vertebrate fauna of the Navesink Formation a large morphotype of ornithomimosaur and a possibly new species of large crocodyliform. Large ornithomimosaurs have also been documented in the Campanian of Mongolia (representing two clades e.g., Osmólska, Roniewicz & Barsbold, 1972; Lee et al., 2014) and Alberta and the Maastrichtian of the United States (“*Struthiomimus*” *sedens*; e.g., Longrich, 2008).

## Conclusions

Partial hindlimb bones of large ornithomimosaurs from the Maastrichtian of New Jersey preserve several types of traces, including those assignable to sharks, a previously undetected morphotype of crocodyliform, and invertebrates. These fossils have the potential to inform taphonomic models for vertebrate fossil deposition in the Atlantic Coastal Plain during that time, evincing the presence of two modes in the Navesink Formation environment. One included taphonomic stages in both near-shore and deep-sea settings, whereas the other was more exclusively marine. Additionally, the specimens add to the diversity of vertebrates in the Maastrichtian of eastern North America, suggesting the presence of large ornithomimosaurs and a potentially unrecognized crocodyliform.

## Institutional Abbreviations

YPM VPPU: Princeton University collection in the Division of Paleontology, Yale Peabody Museum, New Haven, CT, United States

## Additional Information and Declarations

### Data Availability

The following information was supplied regarding data availability: The specimens described in this manuscript are stored at the Yale Peabody Museum in New Haven, CT.
